# Relationship between ambient light and glucose metabolism in healthy subjects

**DOI:** 10.1186/s12868-018-0444-x

**Published:** 2018-07-24

**Authors:** Hirofumi Hirakawa, Takeshi Terao, Koji Hatano, Kentaro Kohno, Nobuyoshi Ishii

**Affiliations:** Department of Neuropsychiatry, Faculty of Medicine, Oita University, Idaigaoka 1-1, Hasama-machi, Yufu-City, Oita 879-5593 Japan

**Keywords:** Ambient light, FDG-PET, Cerebellum, Mood, Glucose metabolism

## Abstract

**Background:**

Given the reported inverse association between light and depressive mood, ambient light may also be associated with some of the brain regions in healthy subjects. The present study aims to investigate the effects of ambient light on glucose metabolism in the brain. We used the data of 28 healthy participants of the no intervention group from our previous randomized controlled trial and analyzed the association between ambient light and [^18^F]-FDG uptake in the brain.

**Results:**

A whole brain analysis revealed a cluster of [^18^F]-FDG uptake that was significantly and inversely associated with log-transformed ambient light in the left culmen of the left cerebellum vermis. After adjustment for age, gender and serum melatonin levels, there remained a significant cluster of [^18^F]-FDG uptake with log-transformed ambient light in the left cerebellar vermis.

**Conclusions:**

The present findings suggest that the uptake of [^18^F]-FDG is significantly and inversely associated with ambient light in the left cerebellar vermis in healthy subjects. The cerebellar vermis may be involved in mood suppression which may be alleviated by light exposure where glucose uptake and metabolism in this area are decreased.

*Trial Registration* This study is a secondary analysis of the previous randomized study which was registered as UMIN000007537. Retrospectively registered (March 20th, 2012).

## Background

A voxel-based meta-analysis of brain metabolism in major depressive disorders demonstrated altered metabolism in the insula, limbic system, basal ganglia, thalamus and cerebellum, suggesting these regions are likely to play a key role in the pathophysiology of depression [1]. Considering the effects of light therapy on not only seasonal but also non-seasonal depression [2], it is possible that at least some of these regions have an association with light in depression. Furthermore, given the reported significant negative association between the illuminance of daylight and depressive mood in healthy subjects [3], ambient light may also be associated with some of the above regions in healthy subjects.

In a previous study, we investigated whether bright light exposure increased [^18^F]- fluorodeoxyglucose (FDG) uptake in the olfactory bulb and/or hippocampus in a randomized controlled trial, which compared 5-day bright light exposure plus ambient light (bright light exposure group, 27 healthy participants) with ambient light alone (no intervention group, 28 healthy participants) [4]. After adjustment for log-transformed ambient light, there remained a significant increase of uptake in the right olfactory bulb [4]. In the present study, as a completely different study, we focused on the no intervention group of the study [4] and analyzed the association between ambient light and [^18^F]-FDG uptake in the brain, in order to investigate the effects of ambient light on glucose metabolism in the brain.

## Methods

### Subjects

Briefly, we used the data of 28 healthy participants of the no intervention group from our previous randomized controlled trial [4] comparing 5-day bright light exposure plus ambient light (bright light exposure group) versus ambient light alone (no intervention group). The Institutional Review Board of Oita University Faculty of Medicine approved the study and written informed consent was obtained from the participants and their anonymity was preserved.

The demographic characteristics of the 28 participants were shown in Table 1. With regard to their mental state; Hamilton rating scale for depression (HRSD) scores, Beck depression inventory (BDI) scores, and Young mania rating scale (YMRS) scores were within normal limits. It should also be noted that more than half of participants had scores of 0 on the HRSD, BDI, and YMRS.

**Table 1:** Demographic characteristics of the 28 participants

Characteristic	
Age, mean (SD, range), years	30.1 (8.8; 20–52)
Gender (M:F)	16:12
HRSD, mean (SD, range)	1.0 (1.5; 0–6)
BDI, mean (SD, range)	1.7 (2.4; 0–9)
YMRS, mean (SD, range)	0.2 (0.5; 0–2)
Season of neuroimaging	Summer 9, winter 9, others 10
Serum melatonin levels, mean (SD, range), pg/mL	19.6 (20.7; 3.9–110.0)
Ambient light, mean (SD, range), lux	397.5 (404.5; 52.0–1787.0)
Log-transformed ambient light, mean (SD, range)	2.4 (0.4; 1.8–3.3)

### The measurement of ambient light, melatonin, and mental state

All participants were monitored by an actigraphy system (actiwatch 2; Respironics Inc., USA) to measure ambient light (including indoor and outdoor light) for 5 days just before positron emission tomography (PET) imaging. Serum melatonin levels were measured just before administering the FDG injection in the morning. Soon after the completion of [^18^F]-FDG injection and PET imaging, all participants were assessed using HDRS, BDI and YMRS.

### Image acquisition

Participants received an intravenous injection of 3.0 MBq/kg of FDG. Scanning was performed for 20 min (matrix = 256 × 256, zoom = 3.0, pixel size = 1.06), and the images were acquired by using a Siemens Biograph PET-computed tomography (CT) scanner (Biograph mCT; Siemens Medical Systems, Erlangen, Germany) with time-of-flight (TOF). Emission data were reconstructed iteratively (OSEM methods, 6 iterations, 21 subsets) and then smoothed with a 2.0 mm Gaussian filter. Data was also corrected for scattering attenuator and decay. No partial volume correction was applied. Attenuation correction was performed using a helical computed tomography.

### Statistical analysis

The association of log-transformed ambient light, HRSD, BDI, YMRS and serum melatonin levels was investigated using the Pearson correlation coefficient. Statistical analyses of the demographic data were performed using IBM SPSS Statistics version 21. Preprocessing and statistical analysis of FDG-PET images were performed using Statistical Parametric Mapping (SPM8) (Statistical Parametric Mapping software, University College of London, London, UK; available at: http://www.fil.ion.ucl.ac.uk/spm/). Anatomic normalization and statistical processing for [^18^F] FDG-PET were performed using SPM8 in conjunction with the Matlab 2014b (The MathWorks, Inc., Natick, MA, USA). The images were normalized into Montreal Neurological Institute (MNI) space and smoothed with an 8 mm full-width at half-maximum Gaussian filter. Proportional scaling to the global mean was used to minimize inter-subject variability. Global uptake differences between brain scans were adjusted using the proportional scaling and individual global counts were normalized to a mean value of 50 mg/100 ml/min. With regard to the whole brain analysis, relative threshold masking was implemented to remove extra-cerebral tracer uptake and 0.8 was used as the threshold. Differences in global activity were removed by proportional normalization of global brain counts to a value of 50. The remainder of the SPM8 default parameters in this module were unchanged.

As a whole brain analysis, multiple regression analysis was performed to assess the association between FDG uptake and log-transformed ambient light. To counteract covariance, we included age, gender and serum melatonin levels as covariates of no interest. The threshold for significance were set at *p* < 0.001 (uncorrected) at voxel level, and *p* < 0.05 (family wise error [FWE] corrected) at cluster level. The results were converted into Talairach Daemon (TD) labels using the Wake Forest University (WFU) Pick-Atlas (Maldjian, Laurienti, Kraft, and Burdette, 2003) for each of the significant cluster was localized and corresponding brain region identified.

## Results

**Fig. 1:**
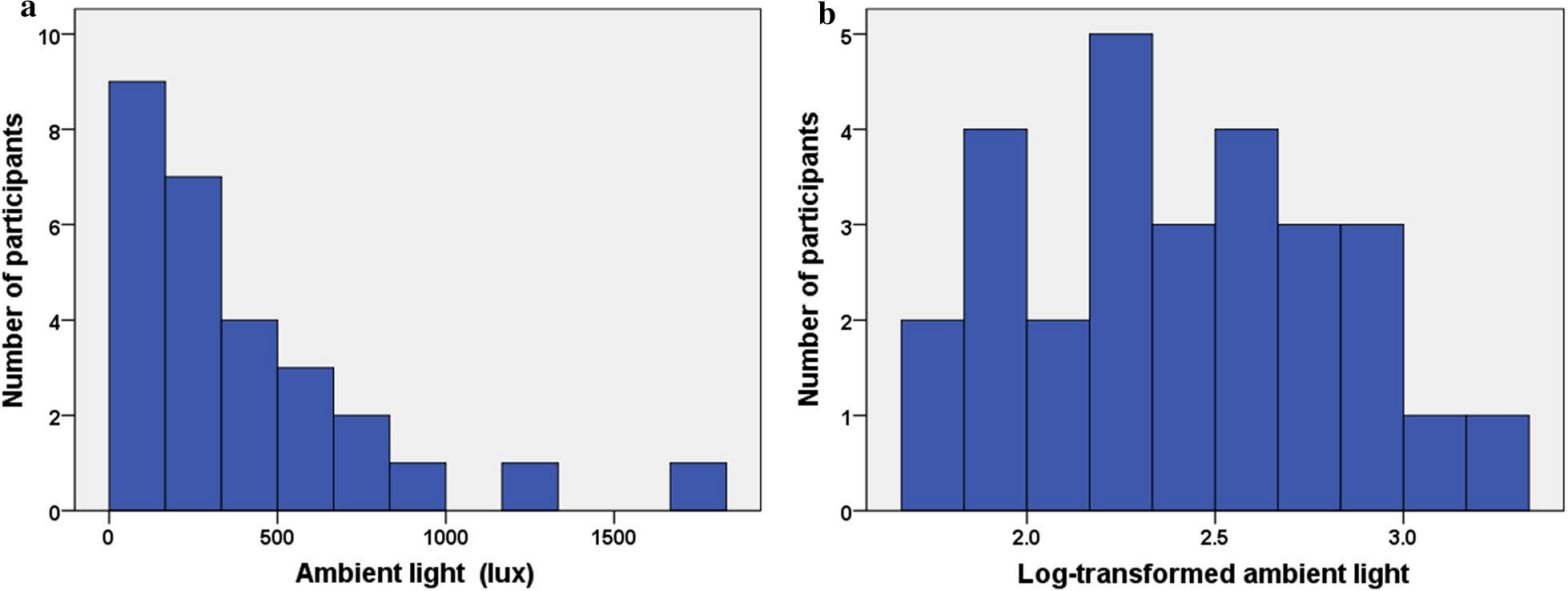
**a** Histograms of ambient light. The distribution of ambient light was considerably skewed to the left. **b** Histograms of log-transformed ambient light. Log-transformation of ambient light was employed in order to use parametric statistical procedures. Log-transformed ambient light showed almost normally distribution

As shown in Fig. 1a, the distribution of ambient light was considerably skewed and therefore log-transformation was employed in order to use parametric statistical procedures (Fig. 1b). Log-transformed ambient light was significantly and negatively associated with serum melatonin levels (r = − 0.38, *p* = 0.04), but there was no significant association between log-transformed ambient light and HDRS (r = 0.23, *p* = 0.23), BDI (r = 0.00, *p* = 0.97) or YMRS (r = 0.16, *p* = 0.39).

**Fig. 2:**
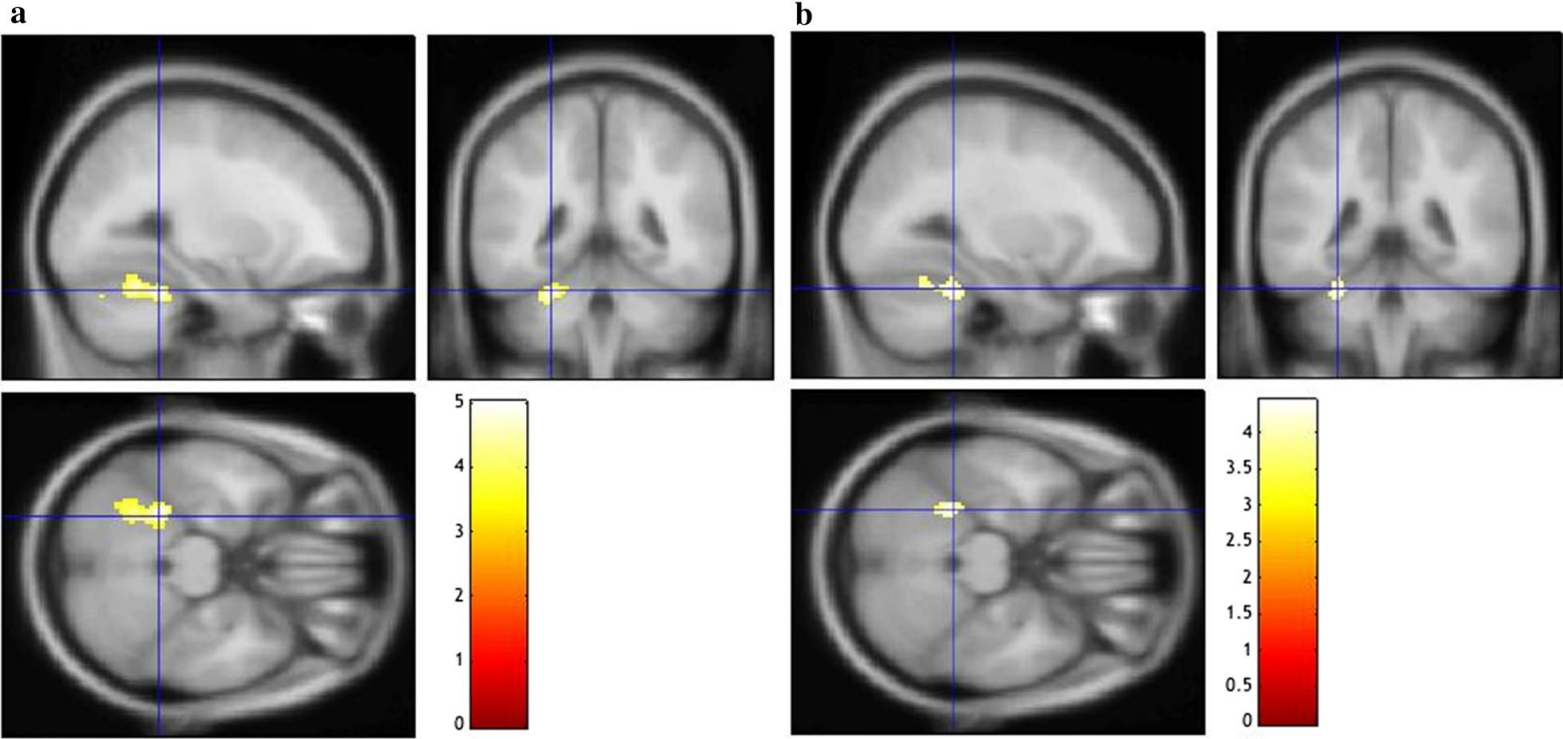
**a** Regions in which cerebral metabolism were significantly and inversely associated with log-transformed ambient light. A whole brain analysis revealed there was a significant reduction of FDG activity with log-transformed ambient light in the left cerebellum, which was overlaid on a T1 weighted MRI template of SPM8. Blue rectangular coordinates indicated a peak level the left cerebellum (MNI coordinates: x, y, z = − 26, − 44, − 26). A color bar shows T value. **b** Regions in which cerebral metabolism was significantly and inversely associated with log-transformed ambient light after adjustment for age, gender and serum melatonin levels. After adjustment for age, gender and serum melatonin levels, there was a significant reduction of FDG activity with log-transformed ambient light in the left cerebellum, which was overlaid on a T1 weighted MRI template of SPM8. Blue rectangular coordinates indicated a peak level the left cerebellum (MNI coordinates: x, y, z = − 28, − 42, − 26). A color bar shows T value

A whole brain analysis revealed a cluster of [^18^F]-FDG uptake that was significantly and inversely associated with log-transformed ambient light in the left culmen of the left cerebellum vermis (Table 2, Fig. 2a). After adjustment for age, gender and serum melatonin levels, there remained a significant cluster of [^18^F]-FDG uptake with log-transformed ambient light in the left cerebellar vermis (Table 2, Fig. 2b).

**Table 2:** Clusters which had a significant association with log-transformed ambient light

	*p* value*	Number of voxels in cluster	Z value of peaks	Coordinates of local maxima (x, y, z) in Montreal Neurological Institute space
Inverse association with log-transformed ambient light				
The left culmen of the left cerebellar vermis	0.00048	346	4.14	− 26, − 44, − 26
Inverse association with log-transformed ambient light after adjustment for age, gender and serum melatonin levels				
The left culmen of the left cerebellar vermis	0.03	157	3.73	− 28, − 42, − 26

Regions labeled by Talairach Daemon of the Wake Forest University Pick-Atlas

*Family-Wise Error corrected *p* < 0.05

## Discussion

The main finding is the presence of a significant cluster of [^18^F]-FDG uptake inversely associated with ambient light in the left cerebellar vermis. This is, to our knowledge, the first finding of its kind and may provide a clue to the effects of ambient light on the left cerebellar vermis. Functionally, the cerebellum is implicated in emotional and behavioral control [5]. Patients with isolated cerebellar disease, particularly those with lesions involving the posterior lobe of the cerebellum and the vermis, commonly present personality change with blunting of affect or disinhibited and inappropriate behavior, which is called “cerebellar-affective syndrome” [6]. Furthermore, acute stimulation of the cerebellum can induce emotional states, including fear and anxiety, whereas chronic stimulation appears to reduce anxiety and depression [7]. Induction of transient sadness in healthy volunteers and patients with depression has been associated with increased cerebral blood flow in the cerebellar vermis [8]. From these findings, it seems likely that there is involvement of the cerebellar vermis in mood and behavior, which probably inhibits mood elevation as suggested by previous studies [7, 8].

On the other hand, as shown in the present findings, ambient light may decrease glucose metabolism of the left cerebellar vermis. Taken together, particularly with the findings in healthy volunteers [8], it is apparent that ambient light decreases glucose metabolism of the left cerebellar vermis which suppress mood elevation, thereby elevating mood. Although there may be other several mechanisms about light [9], the cerebellar vermis may be involved in mood suppression which may be alleviated by light exposure where glucose uptake and metabolism in this area are decreased. This is in line with epidemiological data showing the significantly inverse association of environmental light and suicide [10], which mostly derives from mood disorder.

It should be noted that in the present study, unexpectedly, there was no significant association between log-transformed ambient light and HDRS, BDI or YMRS. This is probably because more than half of the participants had 0 points of HRSD, BDI, and YMRS, and an appropriate association could not be analyzed.

Limitation of this study is that the sample size is relatively small. Also, the definition of different types of lights, the relationship between light and brain metabolism and between glucose metabolism and depressive mood are unclear. Therefore, further studies are needed.

## Conclusion

In conclusion, the present findings suggest that the uptake of [^18^F]-FDG is significantly and inversely associated with ambient light in the left cerebellar vermis in healthy subjects. The cerebellar vermis may be involved in mood suppression which may be alleviated by light exposure where glucose uptake and metabolism in this area are decreased.

## Abbreviations

HRSD: Hamilton rating scale for depression scores
BDI: Beck depression inventory scores
YMRS: Young mania rating scale
FDG: Fluorodeoxyglucose
PET: Positron emission tomography
CT: Computed tomography
